# The dating of leather bindings using ER-FTIR spectroscopy and machine learning

**DOI:** 10.1038/s41598-026-58309-0

**Published:** 2026-06-17

**Authors:** P. Layton, H. Mahgoub, V. Nicoletti, S. Deichstetter, M. Theisen, S. Svoljsak, J. Malešič, B. Spangl, M. Haltrich, M. Strlič, K. Sterflinger, J. Tintner-Olifiers

**Affiliations:** 1 Academy of Fine Arts, Institute of Natural Sciences and Technology in the Arts, Schillerplatz 3, Vienna, 1010 Austria; 2 Heritage Science Lab Ljubljana (HSLL), Faculty of Chemistry and Chemical Technology, University of Ljubljana, Ljubljana, Slovenia; 3 ÖAW, Austrian Academy of Science, Vienna, Austria; 4 NUL, National and University Library of Slovenia, Ljubljana, Slovenia; 5 Department of Natural Sciences and Sustainable Resources, Institute of Statistics, BOKU University, Vienna, Austria; 6 Stift Klosterneuburg, Klosterneuburg, Austria

**Keywords:** Chemistry, Materials science

## Abstract

Libraries and archives hold large collections of medieval manuscripts that are of cultural importance to the regions they serve. These collections are often highly studied for their written material as a snapshot of how life was at the time of writing. By studying that, codicologists can estimate a moderately accurate date for the objects, but it requires significant time, expertise, and effort to achieve these dates. Limitations regarding sample collection and preparation prevent large scale and robust scientific investigation of these culturally significant collections. Recent advancements in the scientific community around non-destructive and non-invasive analysis have helped open the door to study these collections. This study attempts to utilize External Reflectance Fourier Transform Infrared (ER-FTIR) spectroscopy as a tool for molecular decay (MD) dating. By working closely with codicologists and conservators, it is possible to build an MD dating tool by looking at the chemical differences in leather as it ages. With an estimated uncertainty of ± 66 years, this study aims to prove that it is possible to have an accurate dating tool that is fast, easy to interpret, and non-destructive/non-invasive to cultural heritage collections.

**Supplementary Information:**

The online version contains supplementary material available at https://doi.org/10.1038/s41598-026-58309-0.

## Introduction

Leather has been widely used from pre–history to the modern day, valued for its durability and aesthetic qualities. Over the centuries, tanning methods, species used, and preparation techniques have evolved, making leather both a cultural and a technological marker^1,2^. The ability to accurately date leather objects has the potential to help researchers better understand the capabilities and technologies used by the historical bookbinders and scribes. Understanding the age of leather objects provides insight not only into the chronology of manuscripts but also into historical trade, craftsmanship, and material selection.

Libraries and archives have preserved vast collections of dated and undated written materials, many of which retain the original leather bindings. However, these collections are often inaccessible to researchers for scientific dating because of the existing methods, which typically require invasive sampling for techniques such as Carbon-14 dating (C-14) which can achieve an accuracy of up to one year with samples younger than 50 years^3–6^, or the presence of visible wood, in the case of dendrochronology^7,8^. Traditional non-destructive and non-invasive dating approaches require large amounts of time and expertise from codicologists who study decorative toolings, binding structures, and writing styles. Being able to determine the age quickly, and non-invasively would therefore be of significant use for researchers and the institutions who are caretakers of these materials.

Recent advances in infrared spectroscopy and chemometric modelling have opened new avenues for the non-destructive analysis of historical materials. Infrared spectroscopy is a reliable method for detecting the chemical composition of leather^9–18^ without sampling, and has been successfully applied to determine the age of wood^19^ and paper^20,21^. External Reflectance Fourier Transform Infrared Spectroscopy (ER-FTIR) represents a particularly promising approach, offering the advantages of being both non-contact and non-invasive. While the application of ER-FTIR to date leather through molecular decay is new, the results presented in this paper show its potential for reliable dating.

To interpret complex chemical signals related to leather degradation, this study employs machine learning specifically random forest modelling. Random forest models are well-suited for identifying patterns in high-dimensional spectral data and are useful for finding meaning in the chemical structures and their changes during the decay process. The random forest model is considered a black box model where decisions are data-driven and not explicitly defined by the researcher^22^.

By focusing on dating instead of the agents of deterioration, which have been thoroughly studied^13,14,23,24^; this study aims to address this methodological gap by applying ER-FTIR and random forest modelling to a collection of dated historical leathers, thereby evaluating the feasibility of this approach for the future use to non-invasively dating of collections in archives and libraries.

Klosterneuburg Abbey, located 15 min north of Vienna, was an ideal case study for the development of a calibration dataset for leather dating. The abbey was founded in 1114 by Babenberg Margrave Leopold^25^. It had at its founding a scriptorium where manuscripts were produced and bound. This is seen in the ledgers found in the archives that detail materials purchased for the scriptorium. These manuscripts have remained in the library and archives since their creation and have had the same preservation conditions, providing a well-controlled dataset for spectral analysis and modelling.

Establishing a reliable, non-invasive method for dating leather objects has the potential to advance heritage diagnostics and accessibility. By minimizing handling and preserving objects integrity, such techniques align with the goals of preventive conservation while facilitating broader access to cultural heritage materials.

## Results

**Fig. 1:**
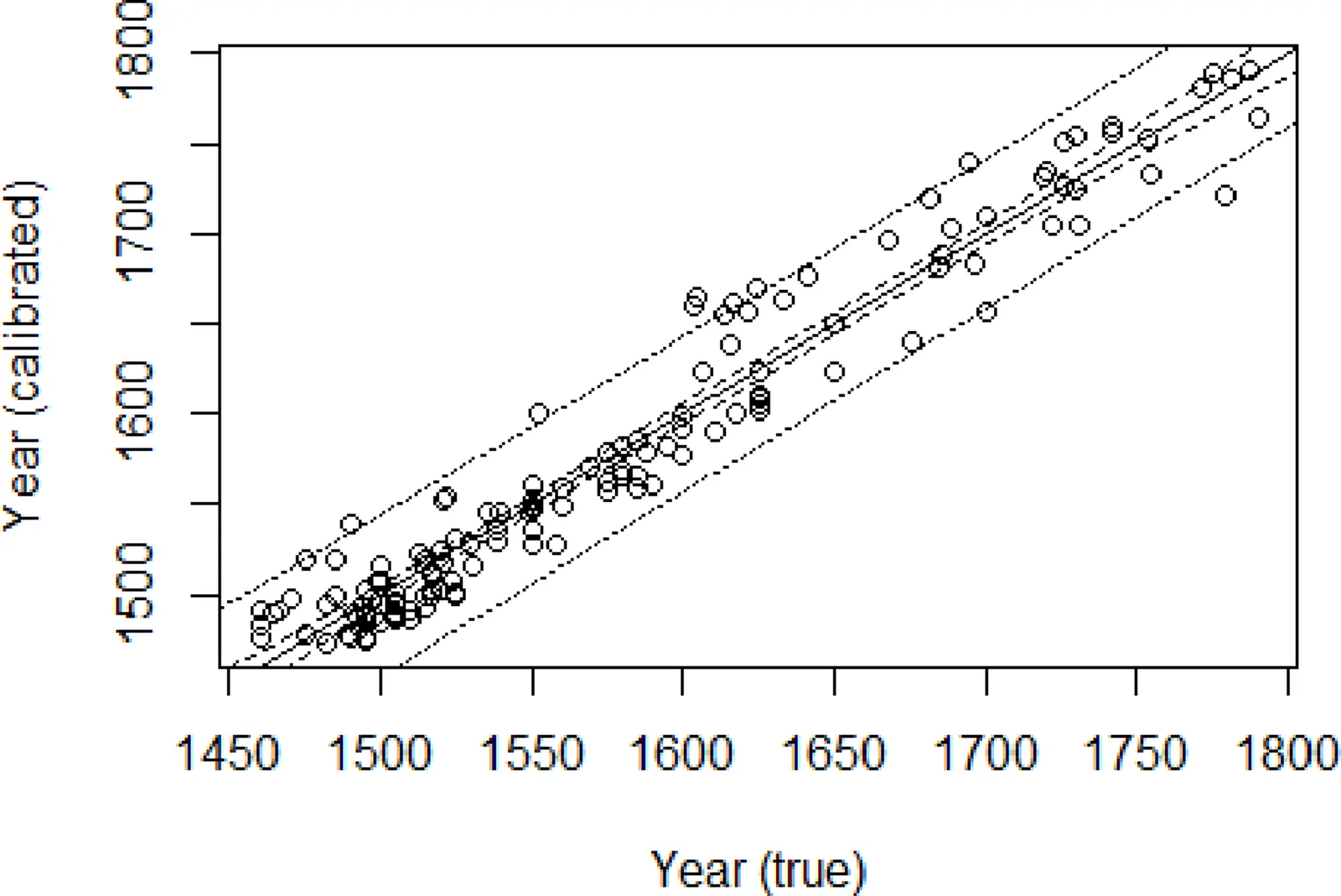
The MD Model plot with confidence and prediction bands. The solid line corresponds to the 45° line, whereas the dashed and dotted lines represent the 95% confidence and prediction bands, respectively. The individual data points, open circles, are the samples (150 incunabula and printed material), the RMSE value for the calibrated data set is: 63.57.

**Fig. 2:**
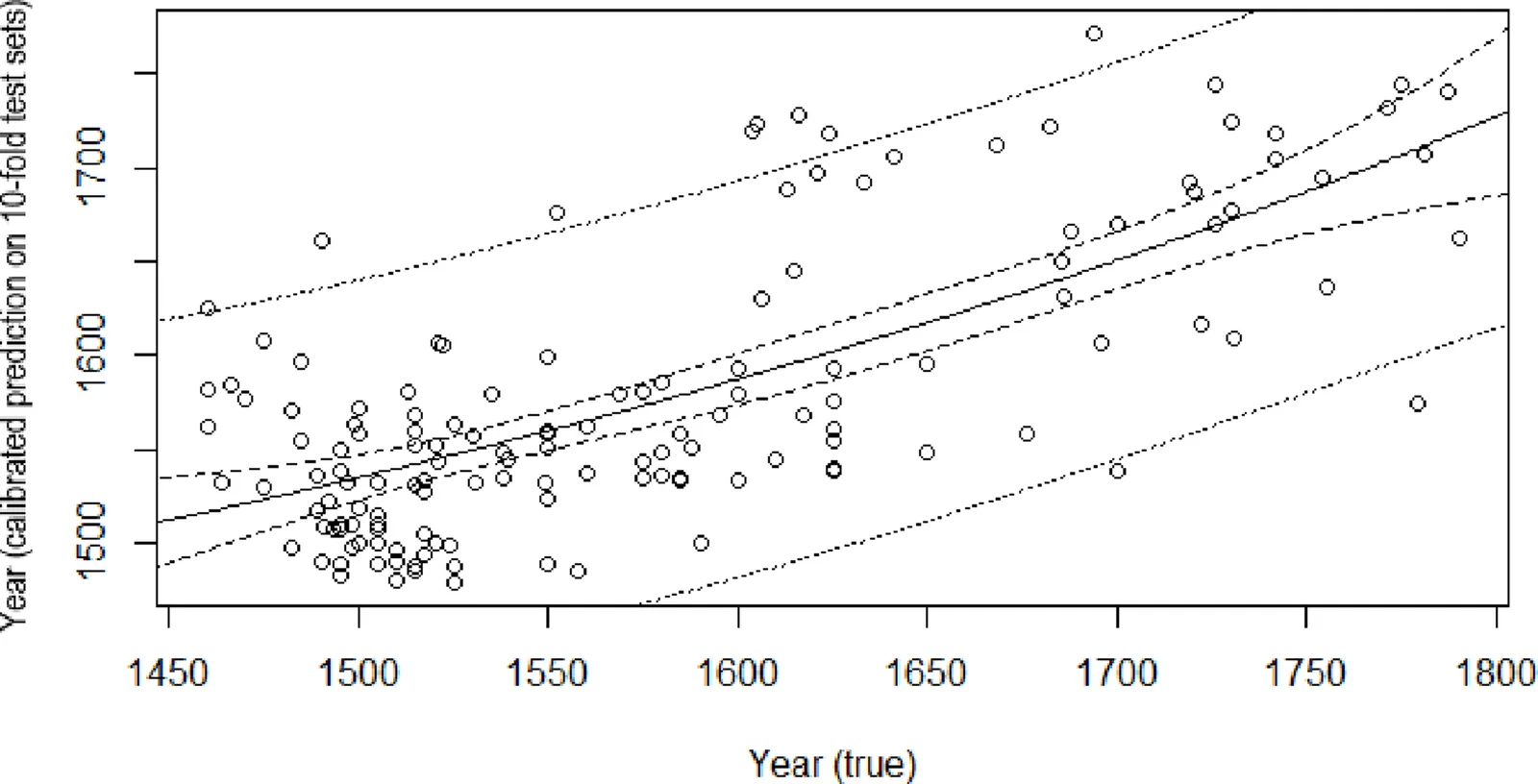
Results of the 10-fold test performed on the calibration set. Positive correlation is retained but a larger deviation of the data points across the graph is better represented. The 10-fold test is performed on the calibrated data set of the initial 150 samples (incunabula and printed material) with a RMSE value of: 63.65.

The developed molecular decay (MD)-model using Random Forest shows a positive correlation between the predicted and known dates of the leather samples (Fig. 1). To explore the influence of spectral range selection, the MD-model was applied to three spectral subsets for testing: Full Range (7500 –400 cm^− 1^), Mid-Infrared (MIR; 4000 –400 cm^− 1^), and Near-Infrared (NIR; 7500 –4000 cm^− 1^). Comparing the models, the MIR range had the highest precision in dating accuracy and the lowest variability across the calibration sets, 10-fold cross-validation tests, and validation sets. Figure 2 shows the 10-fold cross-validation results. Given that the preservation condition is consistent for all the objects in the study, six outliers were identified and removed using Mahalanobis distance within each dataset: Aa II 33, Bc I 681, Bf II 275, Bi I 1246a, Bi I 498, CT 1009.

The root mean squared error of prediction (RMSEP) for the age of leather-bound books from Klosterneuburg was ± 66 years. If the outliers are included in the calculations, the RMSEP increases slightly from ± 66 to ± 68 years. This suggests that, although the Mahalanobis distances flagged these points as outliers, their influence on the overall prediction performance of the model is minimal. Table 1 shows the predictions from the independent validation set of objects from Klosterneuburg using the developed model.

**Table 1:** Actual and Predicted ages of a validation set (15 samples) of objects from Klosterneuburg collection using the Random Forest MD model. The RMSEP of the validation set is ± 66 years which is in line with the prediction model for the calibration set (± 64 years).

Klosterneuburg validation			
ID	Actual year	Predicted year	Diff.
Ab I 28	1728	1631	97
Ac I 49	1687	1665	22
Bb I 175	1550	1539	11
Bb I 177	1687	1626	61
Bb I 490	1683	1663	20
Be II 83	1607	1540	67
Bf I 14	1673	1568	105
Bg III 1021	1550	1521	29
Bg III 110	1560	1533	27
Bh I 100	1665	1550	115
Bi II 82	1571	1540	31
C III 4	1538	1598	-60
L I 10	1776	1715	61
L II 291	1711	1630	81
L II 349	1707	1632	75
	RMSEP: ±65.84		

To evaluate the broader applicability of the model, the calibration set based on Klosterneuburg data was used as the base for a dating model applied to leather-bound samples including 17 from the National Library, Prague, Czech Republic ranging from 1288 to 1763 CE, and 18 samples from NUL in Ljubljana, Slovenia ranging from 1479 to 1800 CE. Although the three libraries/archives maintain similar preservation standards, internal environmental differences may affect leather ageing patterns. Table 2 compares the prediction and actual dates for these external datasets where the RMSEP increased significantly with ± 108 for Ljubljana set and ± 256 for Prague set. Table 1 shows the Klosterneuburg validation set with a prediction of ± 66 years.

**Table 2:** The actual dates and predicted dates from the model are represented here. The material from Ljubljana is on the left and shows a relatively high RMSEP. The RMSEP values are outside the prediction range of the calibration set from Klosterneuburg.

Ljubljana				Prague			
ID	Actual year	Predicted year	Diff.	ID	Actual year	Predicted year	Diff.
GS013829	1548	1611	-63	V F 19	1288	1651	-363
GS0327	1707	1722	-15	XIII D 178	1442	1586	-144
GS04136	1750	1656	94	XIII E 4	1320	1556	-236
GS07042	1743	1568	175	XXIII C 95	1375	1571	-196
GS10257	1770	1705	65	XXIII D 130	1425	1618	-193
GSI10049	1689	1663	26	XXIII D 140	1325	1651	-326
GSI17240	1714	1636	78	XXIII D 180	1475	1606	-131
GSI8119	1767	1671	96	XXIII E 23	1200	1538	-338
GSI9865	1780	1671	109	XXIII E 28	1275	1565	-290
GSII2593	1790	1638	152	XXIII E 30	1150	1609	-459
GSII2601	1795	1637	158	XXIII E 7	1763	1700	63
GSII2646	1775	1667	108	XXIII F 123	1225	1563	-338
GSII2656	1785	1667	118	XXIII F 42	1608	1695	-87
GSII4402	1577	1649	-72	XXIII F 54	1552	1700	-148
GSIII16055	1521	1558	-37	XXIII F 8	1568	1587	-19
GSO10204	1800	1662	138	XXIII G 26	1468	1634	-166
Ti11963	1485	1668	-183	XXIII G 46	1175	1524	-349
TiIII11294	1495	1578	-83				
	RMSEP: ± 108 years				RMSEP: ±256 years		

## Discussion

This study stems from the larger Ancient Book Crafts project^26^ where a combination of intense codicological research and application of ER-FTIR work together to build a dating model.

The random forest model shows the possibility of dating the leather book covers from Klosterneuburg library and archive with a reasonable degree of accuracy based on the codicologically determined ages (Figs. 1 and 2; Table 1). This model builds upon the foundational work of Trafela et al. (2007) and Tintner et al. (2020), who studied molecular decay (MD) in paper and wood^17,19,27–29^. With a non-destructive and non-invasive technique that can be broadly applied in libraries, archives and museums that opens doors and avenues for research into material that have been under studied or inaccessible at all.

This calibration data relies on dating performed by codicologists to set the foundation for the initial prediction range. Analysis by the codicologists looks at differences in handwriting style, ink types, and binding structures^30,31^. As described in the methodology older manuscripts in this study rely on the introduction and retiring of the blind tooled stamps within the abbey. These production aspects and personal styles of the scribes have date ranges that become the starting range for this study. As codicology can obtain an accuracy of ± 50 years, this dating model should not be able to predict more accurately than ± 50 years.

The current model achieves a prediction accuracy of ± 66 years (Fig. 1), which is notable given the complexity and variability of historic organic materials. It shows tendency to underestimate the age of samples suggesting that the calibration set is biased toward older materials. For that, a weighted random forest model was performed with different weight ratios to assess its impact, dividing the weight of the model with a 2:1 and 4:1 weighting on objects younger than 1550 where it shows no impact or improvement on the accuracy of the dating. The amount of available well dated younger objects is limited; older material could be added from the incunabula collection but this would only further skew the data to older predictions. The RMSE breakdown per century can be found in table S1 (supplementary information). Table S1 shows the RMSE per century: 1450–1550 have an RMSE of 71.5, 1550–1659 of 43, 1650–1750 of 67, and 1750–1850 of 122. There are only 8 manuscripts used for the century specific RMSE for 1750–1850 and it is clear that with a low sample group has a large prediction error. Comparing the RMSE of 1750–1850 with that of 1550–1650, which has 45 manuscripts for its sample population, correlates that a larger sample group has better prediction capabilities.

Younger items have the potential for more accurate binding dates, as records of the donators are available and printed dates are often in the text, but there is limited codicological work completed on these objects. The overall material collection for this library is medieval in nature and codicological research has focused on such materials. The model’s estimated dates were closely aligned with the published dates inscribed on the frontispieces of the test data set considering the RMSEP. The difference between the dating of the text block and the leather cover is not unexpected given historical practices where many collectors often rebound materials to suit personal aesthetic or archival preferences^32^.

In an attempt to compare leather covers between separate collections, the ability of the Klosterneuburg model to predict the ages was unsuccessful, see Table 2. Two additional small sample sizes of 10% (Table 2) of the Klosterneuburg dataset were measured and compared to the model (Fig. 1; Table 1).

The results in Table 2 utilized the Klosterneuburg prediction model in an attempt to predict dates for external libraries. Manuscripts from the collections of Prague and Ljubljana were chosen by conservators and librarians, to fit within the date range of the Klosterneuburg model. This prediction model achieved largely inaccurate dates, with the largest spread being 512 years for Prague samples; which is less than ideal for manuscript dating. This discrepancy in dates is due, in part, to the codicological dating of those manuscripts. For example, the dating in Prague focused on the textual material and not the leather covers. This dating method relies heavily on the paleographic and codicological research which when used in conjunction with ER-FTIR MD-dating can help narrow the range of binding date. This large range found in the Prague samples (Table 2) show the limitation of the prediction model and the necessity of strong codicological assessments.

Table 3 depicts chemical bond structures identified by the random forest model. The model itself indicates the regions of importance and how they influenced the model’s predictions. The region of 2980 –2960 cm^− 1^ can be attributed to the Aliphatic C—H stretching^5,12^ that are in the hydrocarbon side chains of amino acids and residual lipids in the leather. The region 1670 –1660 cm^− 1^ are attributed to the conjugated carbonyl double bond also known as the Amide I band^5,12,14^ and is the stretching of the peptide bond. Regions 1575 − 1560 and 1500 –1480 cm^− 1^ are attributed to the N—H bending and the C—N stretching of collagen^5,12,14^. At the lower wavelengths, peaks could indicate the presence of vegetable tannins seen as aromatic rings (C$\:\equiv\:$C) that are part of the tanning process. The regions of 1325 –1300 cm^−1^ and 1200 − 1180 cm^− 1^ suggest that C—O stretching is occurring most likely due to polyphenolic structures of tannins or esters that are bound to the leather’s collagen matrix^33–35^.

**Table 3:** Variance importance plot generated by the random forest MD model. Importance average indicates the effect the wavenumber ranges have on the model’s detection. This simplified table shows the relative importance of each feature spectra with the regional attributes.

Spectral features in cm^− 1^						
	2980 − 2960	1670 − 1660	1575 − 1560	1500 − 1480	1325 − 1300	1200 − 1180
Counts	11	2	3	6	4	4
Importance Avg.	4.8	4.5	3.2	4.3	3.3	3.7

The variable importance analysis from the Random Forest model highlights the C—H stretching band at 2980 –2960 cm^− 1^, with 11 counts averaging with 4.8% of the total variable importance (Table 3), as the primary spectral indicator of molecular decay in leather. This indicates that lipid structures are responsible for the majority of molecular changes associated with leather ageing. Subsequent changes in the amide I (C = O stretching) at 1670 –1660 cm^− 1^ with 2 counts of importance averaging 4.5% intensity, and amide II (C—N stretching) at 1575 –1560 cm^− 1^ with 3 counts of importance averaging 3.2% importance also contribute significantly to the decay but are secondary to the lipid-related features^5,33^. The peaks found in the variable importance plot correlate with the degradation of the tannins and collagen structures in the leather as found by Sebestyén et al. in 2022^15^. While the variable importance list is extensive, it only looks at how the random forest model made its decisions and not at other factors that could impact degradation in a library. Oxidation degradation as the leather ages has a role on the Amide I structure as it has an overlapping vibration at 1750 –1701 cm^− 1^ and the C—H stretching at 2980 –2960 cm^− 1^ may be impacted from contamination due to use or past treatments from consolidation efforts^23^. The spectra collection through ER-FTIR is less sensitive to surface contaminations and is able to get a more average spectra of the leather surface^13,23,24,37^.

The results from the Ljubljana and Prague validation sets revealed limitations in the transferability of the MD model indicating that a different calibration set may be required for each library. Despite all items being stored under typical library conditions, the internal microenvironments of these institutions differ and appear to have influenced the degradation pathways reflected in the spectra as shown in the RMSEP of both external libraries. Prague’s dataset RMSEP is double the error of the Ljubljana dataset. This discrepancy highlights the need to develop localized calibration sets or introduce environmental correction factors for broader model applicability. In the case of Prague library, it is also important to note that the cover material age is unknown, and the model is estimating the age based on the dated interior book blocks. Therefore, it remains unclear whether the discrepancies are due to spectral differences or historical rebinding practices. These results suggest that while the Klosterneuburg-trained model performs well under similar environmental and material conditions, broader applicability will require expanded calibration datasets that account for local material and environmental variability.

Limitations due to collections spaces and their available resources make practical applications difficult. The differences in collection’s climates between libraries, storage conditions, and codicological research impact the results and ability of researchers to collect spectra. Surface contaminants can obscure the intrinsic spectral signature of leather but ER-FTIR does minimize their influence though it does not remove it^37^. To reduce the effect of contamination, treatments, and moisture-related variation coordination with conservators was crucial. Visual examination of the object reveals past treatments for patches, repairs, and staining. Spectra collected away from these areas reduced the influence of the past treatments, and potential moisture damage. To remove areas where the intrinsic leather was obscured by surface contamination, multiple spectra were collected from the whole cover and compared against vegetable tanned leather spectra^10,15,16,18,23^. To remove background humidity a background scan was performed before measuring on every manuscript^37^.

A narrow range of preservation conditions/standards is covered in this model specifically: dry environment typical of libraries. Other preservations conditions would need to be studied in future studies. For example, a damp library environment could potentially accelerate the decay rate, leading to different spectral ageing patterns and necessitating an entirely new model for such conditions. Future studies would benefit from measuring different library collections around Klosterneuburg and the surrounding cities in Austria to see if the model’s applicability is limited to country, region, or geographic conditions. By making the dataset available through Harvard Dataverse, we provide an accessible framework for external researchers to fully replicate our machine learning workflow. Furthermore, this allows future studies to return to the Klosterneuburg Abbey collection to directly compare newly acquired spectra against the baseline data established in this study.

Despite these limitations, the model shows considerable promise as a century-level dating tool. When combined with codicological analysis, it can offer a more complete understanding of manuscript composition and history. In this way, MD models provide a powerful complement to traditional methods, improving chronological resolution and helping researchers interpret the material lives of historical books and documents.

## Methods

### Materials

**Fig. 3:**
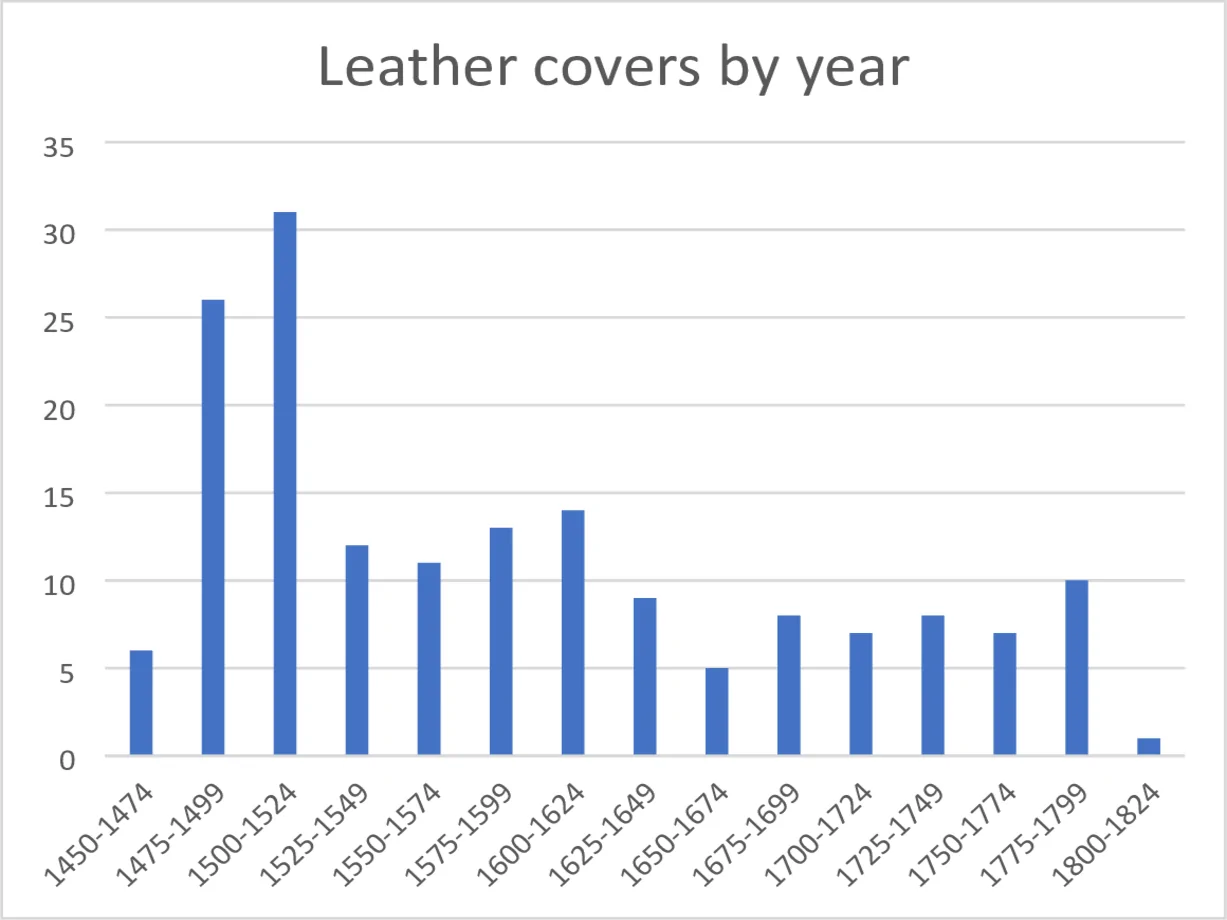
Bar graph showing the bins of dated materials, 150 total samples stretching from 1450–1824. 1830 was determined to be the cut off year to avoid the introduction of chrome tanned leather.

The dataset consisted of 150 leather-bound manuscripts selected from the collections of Klosterneuburg Abbey, Austria (see Fig. 3). These manuscripts span a range of known production dates between 1450 and 1824 AD and were chosen based on the availability of intact leather bindings. Access to the collection was provided through a formal partnership with Stift Klosterneuburg within the grant framework of the Ancient Book Crafts (ABC) project. Detailed information and documentation regarding this research framework can be referenced via DOI https://doi.org/10.55776/I5884. The initial dates were found by the codicologists who are able to distinguish between bindings from the different periods of bookbinding as described by Szirmai^38^. Szirmai’s classification is recognized as the authoritative academic framework for the chronological and regional categorization of medieval bindings. Utilizing this framework, codicologists systematically evaluate physical markers, such as board attachment mechanisms, sewing structures, and covering materials, to accurately identify and isolate gothic-style bindings from the period of the Abbey’s historical bookbinding production. By employing the work “The Archaeology of Medieval Bookbinding,” the ABC codicologists successfully identified gothic-style manuscripts and refined the binding dates by referencing records of stamps found in Klosterneuburg and their usage from manuscript.at, a database that shares motifs from the covers of medieval manuscripts^39,40^. Furthermore, the dating was narrowed by references to the land registers documenting purchases and yearly counts for land holdings of the abbey. This final estimation from all combined codicological assessment was ± 50 years.

The selected volumes have remained within the abbey’s preservation environment since they were acquired or creation, ensuring minimal external degradation influences. The relative humidity (RH) within the collection space of Klosterneuburg has a RH averaging 40 ± 3% per year. Humidity and temperature controls are not present in this library, so the whole spectral collection was performed in winter to have as consistent conditions as possible across samples. The manuscripts were measured on site within the libraries in which they are stored to maintain an equalized environment for measuring within each library.

A prediction model was built using a selection of well-dated incunabula, printed books, and manuscripts from the library and archives of the Klosterneuburg Abbey. The collected infrared spectra were vector-normalized and used to create a calibration set consisting of 3541 measurements from 150 objects. In addition, 300 measurements from 15 separate objects were chosen as a validation set. The validation dataset was kept entirely independent of the calibration training set to ensure an unbiased evaluation. The 15 objects were randomly chosen from the same library collection, utilizing the same historical date range as the calibration set (1450–1824 AD). To ensure data consistency, the baseline requirement for selecting these volumes was the presence of intact, original leather covers with no visual evidence of structural rebinding or modern conservation interventions. The variable importance derived from the Random Forest model highlights which spectral bands play the most significant role in predicting molecular decay, revealing the chemical features driving the model’s performance (see Table 3).

Codicological analysis was conducted on the entire dataset. Watermark analysis, blind tooling stamps, calligraphic record, and binding structure was carried out by the Austrian Academy of Science’s Institute for Medieval Research – IMAFO to provide the initial date ranges for the calibration data set from Klosterneuburg. Similar methods were used for dating in Ljubljana and Prague but by their library staff and conservators.

### Data collection

FTIR spectra were recorded in external reflectance mode using an Alpha II spectrometer (Bruker, Germany). The instrument has a maximum spatial resolution of 2 cm^− 1^. For each measurement, 32 scans were averaged at a spectral resolution of 4 cm^− 1^ and background was collected using an average of 32 scans. Spectra were pre-processed using OPUS software (version 8.4), where the second derivative was calculated followed by vector normalization to enhance spectral features and remove instrumental effects. Spectra were cut into the NIR (7500 –4000 cm^− 1^) and MIR (4000 –400 cm^− 1^) regions. The beam penetration for ER-FTIR is ~ 5 μm and has a sample ellipsis of 1.8 × 2.3mm^41,42^. The beam depth and broad sample size reduces the impact of surface contamination, by following the spectra collection protocol from Layton et al.^37^: spectra for each manuscript were taken 10 times per cover following a simple pattern of 3 locations on the side closest to the spine, 4 locations in the middle, and 3 locations near the fore edge. This gave flexibility to avoid obvious damage, staining, and incompatible spectra. 20 total spectra were taken for each manuscript cover. Visual indication of surface contamination and damage was possible using the built-in camera on the Alpha II, review of the spectra during collection was necessary to remove incompatible spectra and necessitated retaking or repositioning to have the total spectra per cover.

**Fig. 4:**
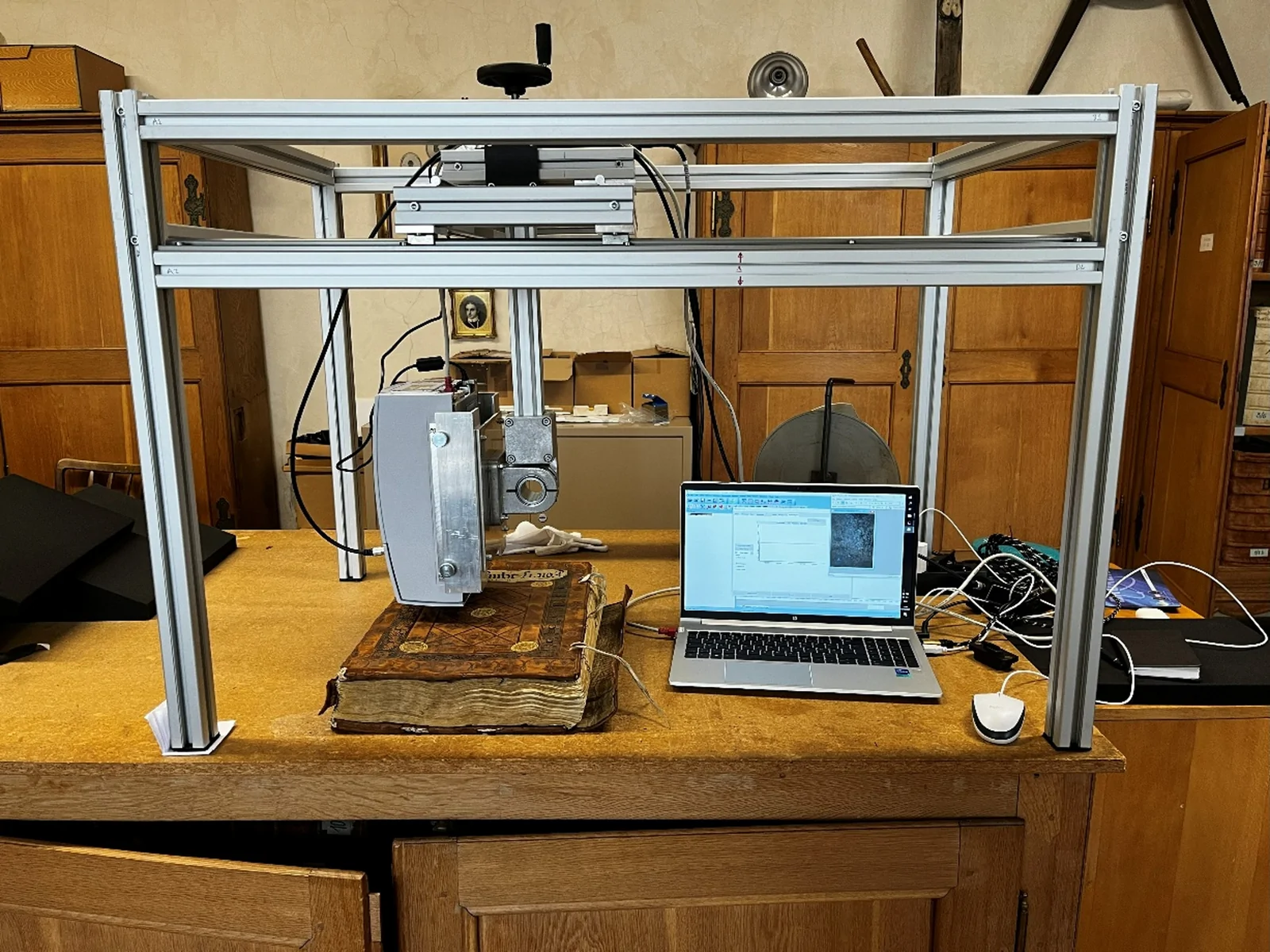
Data acquisition using the Bruker Alpha II in non-contact mode.

To facilitate stable and reproducible measurements, a frame was designed in partnership with the University of Natural Resources and Life Sciences (BOKU) to hold the FTIR instrument above the objects and allow it to move along 3 axes. The design of the frame was based on a quadripod design^43^ but was modified to allow precise, and multi-axis movement for the instrument to move within a grid, see Fig. 4.

### Data analysis

The molecular decay model by Tintner et al.^19^ was adopted to characterize the degradation trends in leather. The model by Tintner et al.^19^ was chosen because it provides a proven, non-destructive mathematical framework for characterizing long-term organic biopolymer degradation through spectral feature extraction. Adapting this model allows us to evaluate whether these established molecular decay pathways share cross-material commonalities with historical leather matrix ageing. The random forest modelling was employed to identify and interpret spectral features correlated with the known initial dates of the samples. All the modelling development was performed in the statistical computer software language R^44^ using the randomforest package^45^ to train and validate the models. A 10-fold cross validation was performed on the calibration set to test the mean accuracy of the model. Results are listed as RMSEP to better understand the calculated ages.

## Supplementary Information

Supplementary Material 1

## Data Availability

Research data supporting this publication is available through Harvard Dataverse: https://doi.org/10.7910/DVN/VOECQK. This data was collected during the FWF project Ancient Book Crafts – ABC and should be appropriately cited.

## Code availability

The random forest code used can be found here: https://github.com/ricolayton/ABC-project. Please refer to the publication: Tintner, J., Spangl, B., Reiter, F., Smidt, E. & Grabner, M. Infrared spectral characterization of the molecular wood decay in terms of age. Wood Sci. Technol. 54, 313–327 (2020), for a more detailed explanation. The code was utilized and adapted with permission from the authors Tintner et al.
