# Supplementary material for: The dating of leather bindings using ER-FTIR spectroscopy and machine learning
Source: Sci Rep. 2026 Jun 17;16:27728. doi: 10.1038/s41598-026-58309-0 (PMC13542212; doi:10.1038/s41598-026-58309-0)
Supplement: Supplementary file 1 [file 41598_2026_58309_MOESM1_ESM.docx]

Supplementary Figures and Tables

| **Century-Specific RMSE (OOB)** | | | |
| --- | --- | --- | --- |
|  | Century | RMSE | Counts (n) |
| 1 | 1450-1550 | 71.50 | 69 |
| 2 | 1550-1650 | 43.22 | 45 |
| 3 | 1650-1750 | 67.50 | 22 |
| 4 | 1750-1850 | 122.14 | 8 |
| **Final Reconciliation** | | | |
| Calculated RMSE | | 67.50 | |
| Actual Model OOB RMSE | | 67.50 | |
| Supplementary Table 1: Calculated RMSE using the original dataset Out-of-Bag prediction compared to the built prediction model. Divided into centuries from 1450-1850. Increased error margins in the 1750-1850 century correlate with a small sample size (n = 8), suggesting a sensitivity to data density. | | | |
